# Next-generation sequencing of the mitochondrial genome of *Bombus longipennis* Friese, 1918 (Hymenoptera: Apidae)

**DOI:** 10.1080/23802359.2021.1976691

**Published:** 2021-09-24

**Authors:** Feng Zhou, Fan Song, Liyuan Yao, Zhibo Hou, Yingzhi Ning, Lingyun Chen

**Affiliations:** a College of Life Science, Northwest Normal University, Lanzhou, China; b Department of Entomology, College of Plant Protection, China Agricultural University, Beijing, China

**Keywords:** Bumblebee, *Bombus longipennis*, mitogenome, phylogenetic analysis

## Abstract

*Bombus longipennis* is the species of Bumblebees (Hymenoptera: Apidae), which are important pollinators for wild plants and greenhouses crops. The complete mitochondrial genome (mitogenome) of *B. longipennis* was determined by next-generation sequencing. The mitogenome was 18,458 bp in size with 87.2% A + T content, containing 13 protein-coding genes (PCGs), 22 tRNA genes, two rRNA genes, and an AT-rich control region (D-loop). Gene arrangement was found to be identical to those of other mitogenomes of bumblebees (e.g. *Bombus terrestris* and *Bombus ignitus*). All 13 PCGs initiated with typical ATN codons. Among them, 11 PCGs terminated with TAA or TAG; only *cox2* and *nad4* have incomplete stop codon T. All 22 tRNAs can be folded into typical cloverleaf structure. Phylogenetic analysis based on the concatenated nucleotide sequences of all 13 PCGs indicated that *B. longipennis* was more closely related to other species of subgenus *Bombus*, which clustered into a monophyletic group.

Bumblebees (Hymenoptera: Apidae) are important pollinators for wild plants and greenhouses crops (Velthuis and van Doorn 2006). The bumblebee group consisted of about 250 known species subdivided into 15 subgenera (Williams et al. 2008). *Bombus longipennis* Friese, 1918 was the species belonging to the subgenus *Bombus* in *Bombus* genus, and widely distributed in North China with elevations of 1303–4011 m (An et al. 2014). Until now, the complete mitochondrial genome of *B. longipennis* has not been reported. Here, we first sequenced and characterized the mitochondrial genome of *B. longipennis* and further tested the phylogenetic relationships combining with other available bumblebee mitogenomes retrieved from the GenBank database.

Female bumblebees of *B. longipennis* were collected from Tianzhu County of Gansu province, China (36°56′35.7″N, 102°30′38.5″E). Specimens were stored in the Institute of Zoology and Ecology, College of Life Science, Northwest Normal University, Lanzhou, China (accession number: TZ2020016). The genomic DNA was extracted from thoracic muscle of a single specimen, and then was sequenced by Illumina NovaSeq 6000 platform with both directions of 150 bp reads. The MITObim v1.9.1 (Hahn et al. 2013) was used to assemble the mitogenome based on 6 Gb clean data. The assembled mitogenome was annotated using the MITOS web server (Bernt et al. 2013) under the invertebrate mitochondrial code. The tRNA genes were confirmed by ARWEN online application (Laslett and Canback 2008). The ClustalX 2.0 software (Larkin et al. 2007) was used to align sequence dataset for phylogenetic analysis. The phylogenetic tree was built using W-IQ-TREE (Trifinopoulos et al. 2016). The newly determined genome from the present study was deposited in the GenBank database (accession number: MW741884.2).

The complete mitogenome of *B. longipennis* was 18,458 bp in length. The A + T content of the whole genome sequence was 87.2% (43.2% A, 44.0% T, 8.4% C, and 4.3% G), indicating significant A + T bias. This mitogenome contained 13 protein-coding genes (PCGs), 22 tRNAs genes, two rRNA genes, and an AT-rich control region (D-loop). The identical pattern of gene arrangement was shared by the mitogenome of *B. longipennis* with other bumblebee species (e.g. *Bombus terrestris* and *Bombus ignitus*) (Cha et al. 2007; Cejas et al. 2020). Like other bumblebees (Du et al. 2016), all the 13 PCGs were initiated with typical ATN codons (four ATG, three ATA, five ATT, and one ATC). Among them, 11 PCGs terminated with TAA or TAG, only *cox2* and *nad4* with incomplete stop codon T. All the 22 tRNA genes, ranging from 57 to 73 bp in length, had the typical cloverleaf structure. The *rrnL* and *rrnS* genes were 1371 bp and 754 bp in length, respectively. The control region was 2418 bp long with 94.0% A + T content.

So far, there are 17 bumblebee species, whose mitochondrial genomes have been reported. These species including *B. longipennis* in this study belong to nine subgenera: *Alpigenobombus*, *Bombus*, *Mendacibombus*, *Megabombus*, *Melanobombus*, *Psithyrus*, *Pyrobombus*, *Sibiricobombus*, and *Thoracobombus*. To confirm the phylogenetic relationships among nine subgenera of the genus *Bombus*, we performed a maximum-likelihood (ML) analysis based on the best-fitting substitution model of TIM + F+I + G4 according to BIC with 1000 ultrafast bootstrap replicates. The concatenated nucleotide sequences of 13 PCGs from 20 Apidae species including geographic populations of *Bombus terrestris* and *Bombus hypocrite* were used to construct the phylogenetic tree. Two stingless bees *Melipona fasciculata* (accession number: MH680930.1) and *Melipona scutellaris* (accession number: NC_026198.1) were used as outgroup for phylogenetic analyses. The result indicated that *B. longipennis* was more closely related to other species of subgenus *Bombus*, which clustered into a monophyletic group (Figure 1). The phylogeny tree also demonstrated a clear relationships among the bumblebee subgenera, which were supported by previous phylogenetic studies of bumblebees (Zhao et al. 2017; Zhao et al. 2019; Wang et al. 2020). The mitochondrial genome of *B. longipennis* reported in this study will provide more essential molecular data for further phylogenetic studies related to bumblebees and other pollinators.

**Figure 1.**
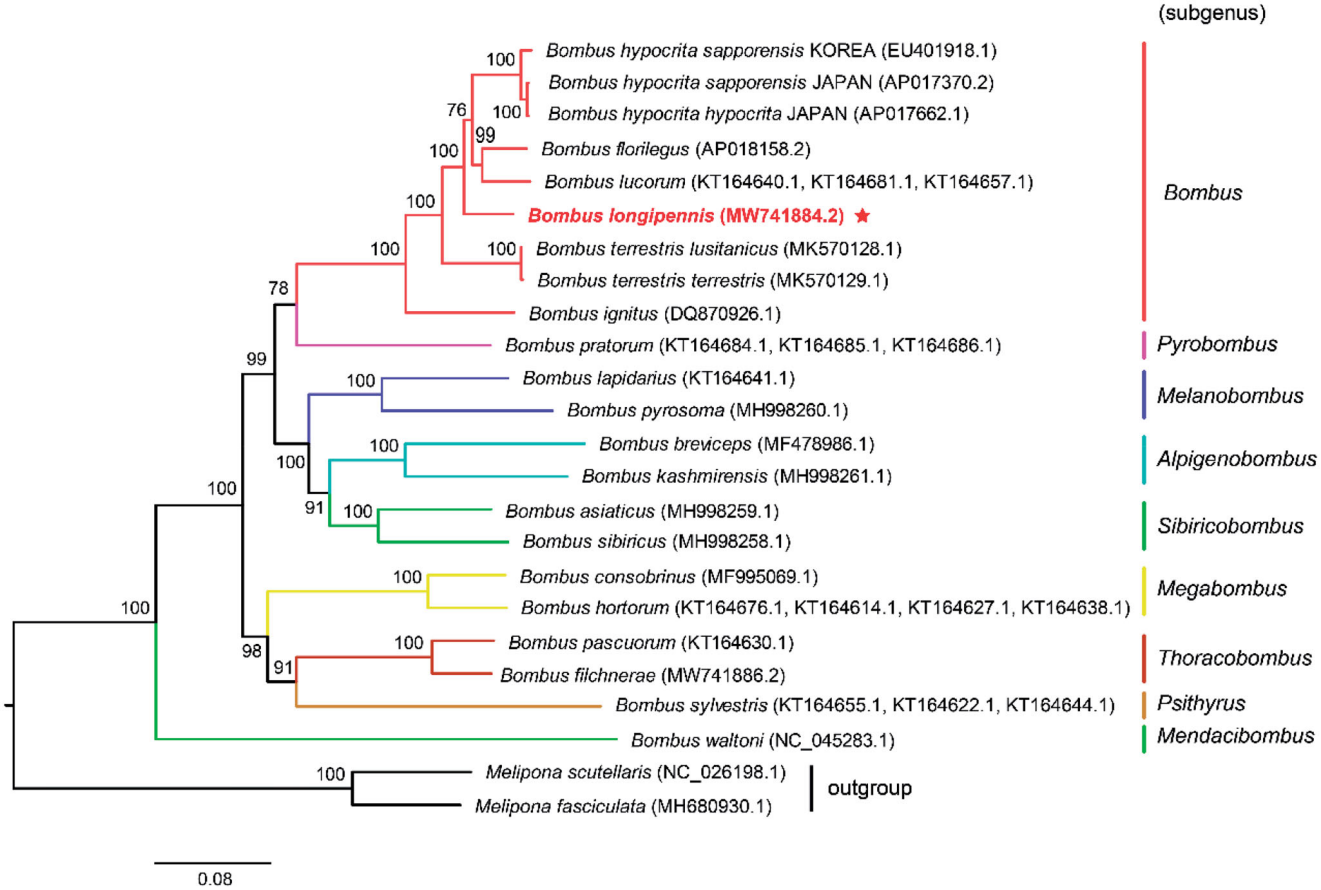
Maximum-likelihood tree showing phylogenetic relationships of *Bombus longipennis* and other 20 Apidae species based on TIM + F+I + G4 model, using concatenated nucleotide sequences of 13 protein-coding genes. Numbers above or below nodes indicated the ultrafast bootstrap support values estimated with 1000 replicates. The subgeneric names and outgroup related to phylogenetic analysis were depicted at right side. The nine subgenera included *Alpigenobombus*, *Bombus*, *Mendacibombus*, *Megabombus*, *Melanobombus*, *Psithyrus*, *Pyrobombus*, *Sibiricobombus*, and *Thoracobombus*. The newly sequenced mitogenome was highlighted by the star.

## Data Availability

The genome sequence data that support the findings of this study are openly available in GenBank at https://www.ncbi.nlm.nih.gov under the accession number MW741884.2. The associated BioProject, SRA, and Bio-Sample numbers are PRJNA707231, SRR13871739, and SAMN18192305, respectively.
